# Functions of pancreatic stellate cell-derived soluble factors in the microenvironment of pancreatic ductal carcinoma

**DOI:** 10.18632/oncotarget.21970

**Published:** 2017-10-19

**Authors:** Qi Wu, Ying Tian, Jingqiu Zhang, Hongpeng Zhang, Fengming Gu, Yongdie Lu, Shengnan Zou, Yuji Chen, Pengxiang Sun, Mengyue Xu, Xiaoming Sun, Chao Xia, Hao Chi, A Ying Zhu, Dong Tang, Daorong Wang

**Affiliations:** ^1^ Medical College of Yangzhou University, Yangzhou, P.R. China; ^2^ Department of General Surgery, Institute of General Surgery, Northern Jiangsu Province Hospital, Clinical Medical College, Yangzhou University, Yangzhou, P.R. China; ^3^ Nanjing Medical University, Nanjing, P.R. China

**Keywords:** transforming growth factor β(TGF-β), interleukin 6(IL-6), galectin-1, stromal cell-derived factor-1(SDF-1), hepatocyte growth factor (HGF)

## Abstract

Pancreatic ductal adenocarcinoma (PDAC) is one of the most lethal forms of cancer with poor prognosis because it is highly resistant to traditional chemotherapy and radiotherapy and it has a low rate of surgical resection eligibility. Pancreatic stellate cells (PSC) have become a research hotspot in recent years, and play a vital role in PDAC microenvironment by secreting soluble factors such as transforming growth factor β, interleukin-6, stromal cell-derived factor-1, hepatocyte growth factor and galectin-1. These PSC-derived cytokines and proteins contribute to PSC activation, participating in PDAC cell proliferation, migration, fibrosis, angiogenesis, immunosuppression, epithelial–mesenchymal transition, and chemoradiation resistance, leading to malignant outcome. Consequently, targeting these cytokines and proteins or their downstream signaling pathways is promising for treating PDAC.

## INTRODUCTION

Pancreatic ductal adenocarcinoma (PDAC) comprises 90% of pancreatic cancers, which is one of the most malignant cancers in the world. The 5-year survival rate of patients with PDAC is < 5% and is only < 20% in patients who undergo curative resection [1]. The reason for poor prognosis is mainly due to that most patients are diagnosed at an advanced stage, when the tumors are considered unresectable, and most of them have a chemoradiation resistance profile [2]. There are increasing attentions on the tumor microenvironment (TME), owing to the vital role it plays in PDAC progression [3]. Histologically, the percentage of malignant cells is much lower than the stroma in pancreatic cancer which forms a desmoplastic fibrotic network containing immune cells, endothelial cells, cancer-associated fibroblasts, pericytes, and pancreatic stellate cells (PSCs) [3–6].

PSCs were first discovered in 1982 in mouse pancreas, which stored lipid droplets containing vitamin A [7]. In 1991, PSCs were identified in healthy rats and humans [8]. In 1998, PSC isolation and culture techniques were invented [9], followed by rapid development of study in the mechanisms of PSC function in the progression of PDAC. However, the origin of PSCs is still controversial. Previously, it was believed that PSCs were derived from the neuroectoderm, but recent studies have reported that PSCs could have a mesodermal origin, as PSCs and hepatic stellate cells have similar biological features [10]. We now know that most proliferating PSCs are from resident PSCs in the pancreas; however, some studies have demonstrated a different source: bone marrow [11]. Furthermore, infiltrating monocytes can be converted into PSCs under specific conditions, e.g., following stimulation by monocyte chemoattractant protein-1 (MCP-1) [12]. Therefore, clarifying the actual source of PSCs could be helpful for PSC-targeting treatment, however, there is no evidence to support that PSCs from different sources have different functions or can be identified and targeted specifically in PDAC and it needs to study further in the future.

**Figure 1:**
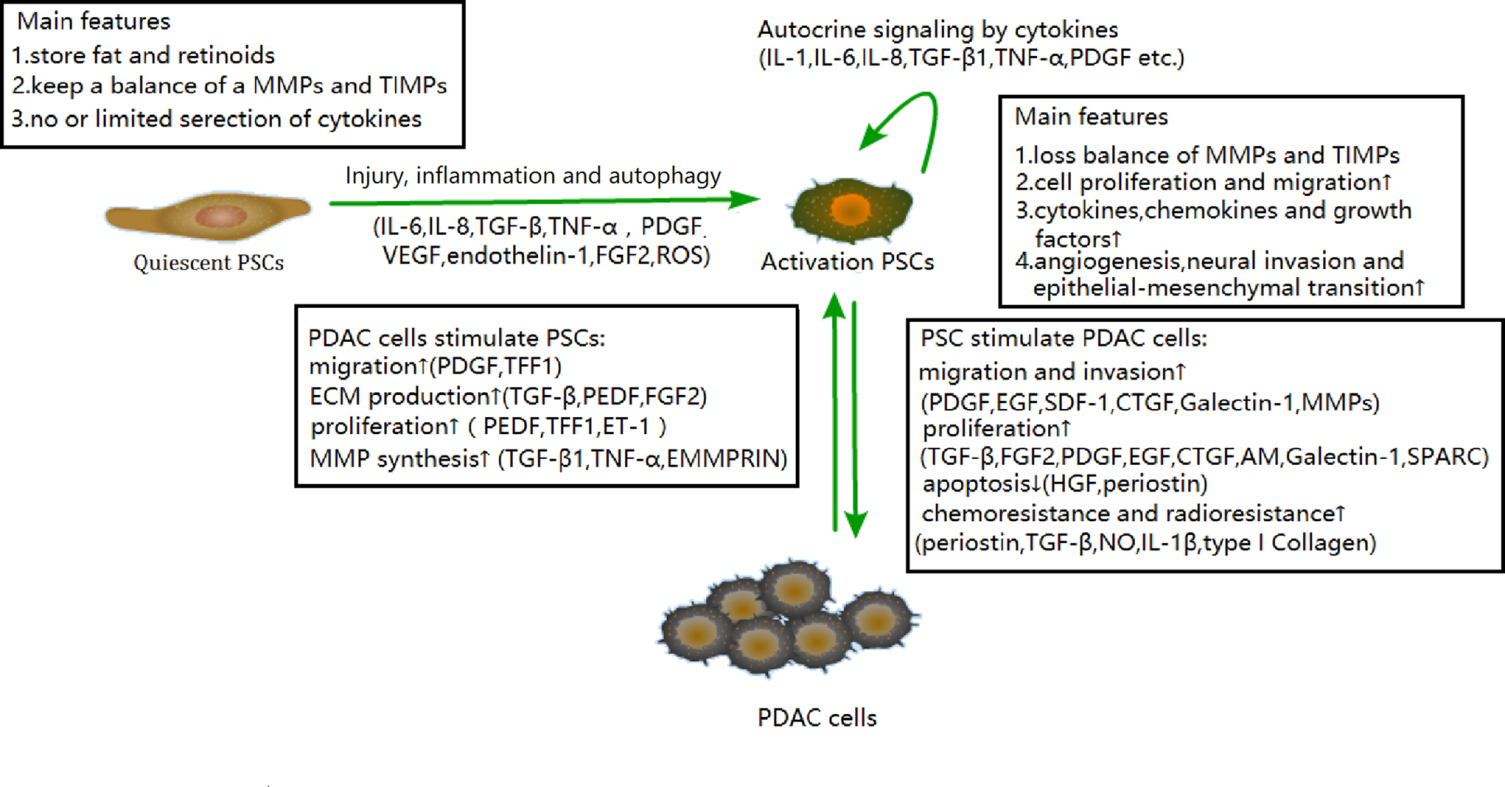
Various features of quiescent PSCs and activated PSCs Activated PSCs can regulate PDAC cell migration, invasion, proliferation, apoptosis, chemoresistance, and radioresistance. Conversely, PDAC cells can stimulate PSC migration, ECM production, and MMP synthesis, which forms a positive loop based on cytokines secreted by PSCs or tumor cells in TMEs. PDGF, platelet-derived growth factor; EGF, epidermal growth factor; CTGF, connective tissue growth factor; AM, adrenomedullin; SPARC, secreted protein acidic and rich in cysteine; HGF, hepatocyte growth factor; NO, nitric oxide; PEDF, pigment epithelium-derived factor; TFF1, trefoil factor 1; ET-1, endothelin-1; EMMPRIN, extracellular MMP inducer.

Generally, there are two main PSC statuses: quiescent and activated. Most quiescent PSCs store fat and retinoids in perinuclear droplets and express glial fibrillary acidic protein (GFAP). And the quiescent phenotype of PSCs could be defined via presence of lipid droplets in the cytoplasm [8, 13, 14]. Quiescent PSCs play a role in maintaining ECM growth and keeping the balance between secreted matrix metalloproteinase (MMP) and tissue inhibitors of MMP (TIMP). Some have argued that quiescent PSCs may also have immune and intermediary functions [14–17]. Quiescent PSCs could be activated in harmful conditions such as stress or inflammation, and the activation was demonstrated to be related to autophagy [18], an alternative metabolic pathway allowing tumor cells to obtain energy [19]. In addition, a recent study found that PSC activation levels could vary according to the microenvironment [20]. The typical features of activation are the expression of α-smooth muscle actin (α-SMA) and a significant quantity expression of ECM proteins (including collagen, laminin, fibronectin) [21]. In PDAC, the activation of PSC contributes to improved tumor cell initiation, development, evasion of immune surveillance, invasion, metastasis, and resistance to chemoradiation [22–24]. Moreover, activated PSCs can produce soluble factors such as transforming growth factor β (TGF-β); interleukin 1, 6, 8 (IL-1, IL-6, IL-8); stromal cell-derived factor-1 (SDF-1); hepatocyte growth factor (HGF), galectin-1 etc. to participate in PDAC cell formation and malignant behaviors. Chronic pancreatitis (CP), which is characterized by massive fibrosis, was demonstrated to be the potential precursors of PDAC [25, 26]. Previous study illustrated that fibrosis is the common feature of PDAC and CP, and PSCs are responsible for the promotion and maintenance of fibrosis [27]. Some PSC-derived cytokines such as TGF-β and fibroblast growth factor (FGF) could contribute to the synthesis of ECM, which leads to the development of fibrosis in CP [28]. Moreover, TGF-β, FGF, platelet-derived growth factor (PDGF) were demonstrated to contribute to the malignant transformation in CP [29], and that could be the reason for carcinogenesis of activated PSC in CP. A recent study argued that TGF-β, FGF and interleukin could activate autophagy in PDAC cells [18], and autophagy dependent alanine secreted by PSCs played a role in pancreatic cancer metabolism. This autophagy process was proven to be stimulated by PDAC cells in turn [30]. Furthermore, PDAC cells can interact with PSCs via the similar soluble factors, stimulating the PSC inflammatory profile, proliferation, and ECM and MMP synthesis, forming a vicious cycle [27, 31] in PDAC (Figure 1).

### The role of PSC-derived TGF-β in PDAC malignant progression

In PDAC, TGF-β is a key signaling mediator involved in stroma–tumor cross-talk, epithelial–mesenchymal transition (EMT), and tumor invasion, in addition, TGF-β was found to be produced by PSCs in the tumor stroma [31–33]. In the classic TGF-β/SMAD signaling pathway, TGF-β combines with its receptor in PSCs, and the activated receptor phosphorylates SMAD2/SMAD3, which combines with SMAD4 and this combination will be translocated to the PSC nucleus [34]. Then, PSCs produce ECM proteins, i.e., collagen, which can promote desmoplastic stroma in PDAC. However, its functions in tumor stroma are various and its characteristics depend on the microenvironment [35, 36]. In non-neoplastic epithelium, TGF-β can be a potential tumor growth suppressor; but in advanced cancer, TGF-β can be a tumor promoter, and this paradoxical switch during tumorigenesis has been linked to EMT process [37, 38]. TGF-β could inhibit stroma related cancer progression, and it could also induce proliferation and migration of pancreatic cancer cells [39, 40]. Here, we discuss the actual mechanistic basis of the novel functions of TGF-β, which could provide ideas for treatment targeting TGF-β or its related signaling pathways in PDAC.

### The role of TGF-β in EMT in PDAC

EMT is a developmental process wherein the cell phenotype shifts from epithelial to motile, fibroblast-like morphology [41], which has been widely studied in various field such as tissue fibrosis and cancer progression [42, 43].

Tumor cells undergoing EMT have reduced intercellular adhesion, with decreased E-cadherin expression or β-catenin translocation and increased expression of mesenchymal markers such as vimentin, fibronectin, and N-cadherin [41], which is reversible and is typically believed to promote invasiveness, metastasis, resistance to chemotherapeutic agents, and sometimes, EMT could induce the emergence of cancer stem cell (CSC) phenotypes in cancers including PDAC [36, 41]. As the first identified cytokine that induces EMT in PDAC, much attention has been paid to PSC-derived TGF-β recently. There are several known TGF-β signaling pathways involving EMT in PDAC, dividing into SMAD-dependent and SMAD-independent signals such as the novel NADPH oxidase 4 (NOX4)-derived reactive oxygen species (ROS) signaling [44] and the cross-talk with Ras/Rad/ mitogen-activated sprotein kinase [45]. The repressors of these pathways are promising for treating PDAC.

TGF-β/SMAD pathway is a predominant promoter of EMT [46]. It is traditionally illustrated that TGF-β could be a growth suppressor in early-stage cancer and typically function as a tumor promoter in advanced cancers [36]. Interestingly, David et al. demonstrated that the PDAC cell phenotypes depend on whether SMAD4, a common transactivator protein, is present or absent [47]. The authors clarified that, in SMAD4-positive tumor cells, the SMAD2/3/4 complex can induce the expression of EMT-associated transcription factors such as SNAIL, which contributes to EMT. On the other hand, it can repress another transcription factor, Krüppel-like factor 5 (KLF5), the presence of which can promote SMAD2/3 pathway leading to tumor progression. In the absence of KLF5, the same SMAD2/3 pathway can cause PDAC cell apoptosis, which can repress tumor development. This could explain why loss of SMAD4 expression is generally associated with worse prognosis [48]. However, it remains to clarify whether EMT initiated by other ligands in SMAD4-positive cells would similarly lead to apoptosis. As mentioned above, it is easy to surmise that small-molecule inhibitors KLF5 could become another anti-metastatic agent in PDAC.

MicroRNAs (miRNAs), small, noncoding RNAs that target mRNAs, have become a current cancer research hotspot. There are some valuable findings about miRNAs in relation to EMT in PDAC, and they could regulate the progression of this cancer [49]. Their downstream targets determine the miRNA function. Some miRNAs were involved in regulating TGF-β-induced EMT, e.g., the miR-200 family (miR-200a, miR-200b, miR-200c), which is downregulated in cells that have undergone TGF-β-induced EMT, and EMT was prevented by their artificial expression [50]. Table 1 lists several recent findings on TGF-β-related miRNAs that could play a role in regulating EMT in PDAC.

**Table 1:** MicroRNAs involved in TGF-β-induced EMT in PDAC

MicroRNA	Target	Functional role
miR-367 [139]	SMAD7	High level miR-367 downregulated SMAD7 expression and involved with poor progronsis in PDAC; miR-367 promoted EMT by increasing the expression of TGF-β
miR-10b [140]	Tat-interacting protein 30 (TIP30)	MiR-10b overexpression accelerated PCC proliferation and tumor growth; miR-10b enhanced the stimulatory effects of EGF and TGF-β on cell migration and EMT, decreasing the expression of RAP2A, EPHB2, KLF4 and NF1.
miR-323-3p [141]	SMAD2 and SMAD3	Silencing of miR-323-3p increased the migration and invasion of PDAC cells, which could be promoted by loss of inhibition of TGF-β-induced EMT; low levels of miR-323-3p predicted poor prognosis in patients with PDAC.
miR-663a and miR-4787-5p [142]	not clear	Lentiviral overexpression of miR-663a and miR-4787-5p reduced TGF-β1 synthesis and secretion in PDAC cells, which presented a EMT-resist phenotype just like being stimulated by 3-deazaneplanocin-A (DZNep).
miR-655 [143]	ZEB1 and TGFBR2	Researchers used a reporter system based on a stable clone derived from a pancreatic cancer cell line (Panc1) and they found that overexpression of miR-655 could upregulate E-cadherin and downregulate typical EMT-inducers accompanying the suppression of migration and invasion of mesenchymal-like cancer cells.

As mentioned above, PSC could induce the EMT process of cancer cells [51], however, Tian L et al. recently demonstrated that EMT is a vital process during PSC activation as well, followed by significant alterations in migration, morphology capacity, and the expression of EMT-related gene *in vitro* [52]. Bone morphogenetic protein 7 (BMP7), a TGF-β-induced EMT antagonist [53], was a positive regulator of mesenchymal–epithelial transition (MET, the reverse of EMT) and was significantly decreased in mice of chronic renal injury [54], which was demonstrated in prostate and breast cancer cells too [55, 56]. In breast cancer, therapeutic administration of BMP7 could diminish breast cancer metastasis to bone [56]. Accordingly, BMP7 could be a promising antagonist to induce MET to restore quiescence in activated PSCs, which would be a potential therapeutic strategy for pancreatic cancer.

### TGF-β and chemoradiation resistance in PDAC

Chemoresistance is one reason for the poor prognosis of PDAC, and this is a major problem during the treatment of this lethal cancer as well. Unfortunately, even well-known first-line agents, i.e., the existing standard gemcitabine therapy, have little effect and can only modestly prolong survival [57, 58].

Compared with other canonical cytokines contributing to chemoradiation resistance, such as IL-1β and nuclear factor-κB (NF-κB), PSC-derived TGF-β is a novel cytokine involved in the promotion of chemoradiation resistance in PDAC [27]. As discussed above, TGF-β participates in EMT process, which is linked with CSC development [59]. CSCs are considered a determining factor in chemoresistance and radioresistance, and they are enhanced by PSCs [60]. In fact, TGF-β could promote the CSC development, and this was proven in PANC-1 and PSN-1 pancreatic cancer cell lines. The researchers found that tumor cells were sensitized to radiation via the inhibition of EMT and CSC process following the use of multi-dose TGF-β-neutralizing antibody. Therefore, we may conclude that PSC-derived TGF-β expression in TMEs could play a vital role in PDAC chemoradiation resistance, and this effect might be based on TGF-β-induced EMT and CSC process. We have discussed that ECM proteins produced by PSCs are a considerable part of the desmoplastic stroma in the PDAC microenvironment, and they play an important role in initiating stromal–cancer cell cross-talk and limit chemotherapeutic drugs’ delivery and effectiveness, inducing chemoresistance. As TGF-β is one of the most important factors stimulating PSC secretion of ECM [61], more attention has been focused on it recently. There is cross-talk between TGF-β and Sonic Hh (SHH) signaling, another pathway involved in PDAC chemoresistance [62]; to be exact, the SHH pathway can be the downstream signaling pathway of TGF-β during pancreatic fibrosis, which can be the precursor to PDAC [27, 63]. Kenneth P. Olive et al. found that inhibiting SHH pathway could enhance the effectiveness of gemcitabine for a short term in mice [2]. However, recent clinical trials demonstrated that SHH inhibitors(such as vismodegib; Genentech, South San Francisco, Calif) combined with gemcitabine yielded no significant improvement on progression or overall survival, even increased mortality [64, 65]. Nevertheless, novel mechanisms such as autophagy [66, 67], and novel pathways involving TGF-β have been found recently, providing new ideas for overcoming chemoresistance in PDAC (Table 2).

**Table 2:** TGF-β-mediated agents or potential targets for chemoresistance in PDAC

Agent or potential target	Descsription	Mechanism of action	Influence on PDAC
Ormeloxifene [58]	a nonsteroidal triphenylethylene compound	blocked the Hh signaling pathway by inhibiting the important effectors of this pathway, such as SHH, SMO, Gli-1, and SDF-1 (CXCL12) , inhibited desmoplasia and interrupted the tumor–stromal interactions.	reduced tumor progression, invasion, metastasis, and chemoresistance and enhanced the antitumor effect of gemcitabine.
Lumican [144]	an extracellular matrix proteoglycan secreted by PSCs	can significantly decrease AMP-activated kinase (AMPK) activity, inhibiting chemotherapy-induced autophagy in both *in vitro* and *in vivo*. TGF-β can negatively control lumican transcription in PSC through novel SMAD4-SBE binding.	improved the cancer cells’ sensitivity to gemcitabine, which was reflected in increased mitochondrial damage, reactive oxygen species (ROS) production and cytochrome C release.
Simvastatin [145]	a member of Statins family	attenuated the tumor-associated macrophages(TAM)-mediated gemcitabine resistance of PDAC by blocking the TGF-β1/Gfi-1 axis.	functioned on preventing chemoresistance in PDAC and was reported to reduce 67% risk of pancreatic cancer.
CYR61 [57]	a matricellular protein, cysteine-rich angiogenic inducer 61	TGF-β induced the expression of CYR61 in PSCs through canonical TGF-β-ALK5-SMAD2/3 signaling.	increased cellular uptake of gemcitabine and sensitize PDAC cells to gemcitabine-induced apoptosis.
TAK1 [146]	TGF-β-activated kinase-1, a mitogen-activated protein kinase kinase kinase	increased the NF-kB- and AP-1-mediated transcription of cIAP-2.	suppressed proapoptotic signaling pathways, resulting in chemoresistance in PDAC.
miR-17-92 [147]	a member of miRNAs	inhibited NODAL/ACTIVIN/TGF-β1 pathway, which could increase cancer stem cells(CSC)’ chemoresistance.	abrogated CSC phenotypes and their tumourigenicity in PDAC.

### PSC-derived TGF-β and Immunotherapy: combination medication and personalized treatment

Most of PDAC contents are ECM and non-neoplastic cells such as PSCs, which play a critical role in PDAC development; as a result, many tumor cell-targeting treatments fail to eradicate PDAC [68, 69]. However, simple removal of stromal cells may lead to immunosuppression and shortened survival of patients, because stromal cells have inhibitory effects on pancreatic cancer progression simultaneously [70, 71]. Therefore, understanding the underlying molecular mechanism could help researchers to seek suitable therapies involving immunotherapy for patients with PDAC.

The human immune system has protective factors against illness which can identify ‘enemies’ and create suitable ‘weapons’ for defense. This ingenious system includes CD8+ cytotoxic T cells, macrophages, dendritic cells, and natural killer cells [72]. However, multiple mechanisms remarkably suppress these tumor-specific immune responses in malignancies such as PDAC, even in the early stages, and this process including TME infiltration by immunosuppressive cells such as myeloid-derived suppressive cells (MDSCs), tumor-associated macrophages (TAMs), and regulatory T cells (Tregs). PSC-derived TGF-β could likewise play a role in immunosuppression via inhibiting the above immune cells and increasing Tregs’ numbers [73]. TGF-β could induce the expression of the transcription factor forkhead box 3 (FOXP3), which can be a determinant factor in Tregs’ development, and the Tregs can secrete immunosuppressive cytokines that can suppress CD8+ T cell killing of tumor cells [74].

Using low-dose cyclophosphamide could downregulate TGF-β-induced Tregs’ number and functionality. However, this therapy could have inevitable adverse effects because it could do harm to the tumor-suppressive immune cells as well [75]. Given the multiple factors and various conditions in tumor progression, combination medication has its edge in some contexts. Interestingly, Malvicini et al. found that combining subtherapeutic doses of an adenovirus expressing IL-12 gene and low cyclophosphamide doses could inhibit IL-10 and TGF-β, modifying Treg performance and prolonging the survival of pancreatic carcinoma in an animal model [76]. Similarly, Soares et al. found that TGF-β inhibition combined with a allogeneic pancreas tumor vaccine secreting granulocyte/macrophage colony–stimulating factor (GM-CSF) dramatically increased effector CD8+ T lymphocyte infiltration and inhibited Tregs more significantly, followed by a survival advantage in a mouse trial [73]. As the vital role TGF-β plays in immunosuppression, combining TGF-β inhibitors such as the small-molecule galunisertib [77] or other repressors targeting downstream of TGF-β signaling with existing immunotherapy strategies could be more effective for PDAC treatment.

Based on the individualized difference of gene mutations, TGF-β-inhibiting therapy for each patient with PDAC could have different effects, therefore, it is meaningful to implement personalized treatment in PDAC patients. A recent study has shown that the loss of transcriptionally active p73 (TAp73), a p53 family member, could be a determinant factor for selecting patients who can benefit from TGF-β-inhibiting therapy [78]. This study displayed that the absence of p73 was involved in TGF-β signaling activation through a SMAD-independent pathway, followed by oncogenic effects such as EMT induced by TGF-β; in the presence of TAp73, the opposite effects were observed. However, eligible patients for TGF-β-inhibiting therapy are restrained at present [78] and the lack of more precise selection biomarkers for patient populations is the main barrier to its practice [79].

### Roles of SDF-1 in PDAC invasion and metastasis

Chemokines are a small chemotactic cytokine family. SDF-1 (or, CXCL12) is a chemokine expressed in certain cancers, involved in tumor cell migration and metastasis [80, 81]. In particular, the SDF-1/CXCR4 axis, which was demonstrated by many recent studies, plays a crucial role in tumor–stromal interactions [82]. In PDAC, PSC-derived SDF-1 could promote PDAC cell invasion and gemcitabine chemoresistance by virtue of this axis [83, 84]. TAM recruitment to the tissue via CXCR4 expression in response to SDF-1 is an essential process of cancer initiation, formation, progression, and migration [85]. Particularly, in tumor metastasis, the formation of the pre-metastatic niche was recently established, and was considered the metastatic cancer prodromal stage [86]. It was recently proven that SDF-1 is involved with this process. In a murine model of Lewis lung carcinoma, SDF-1 was elevated prior to cancer cell infiltration to the lymph nodes, and inhibition of this SDF-1 signaling axis (cyclooxygenase 2/prostaglandin E receptor 3-dependent induction of SDF-1) is promising for suppressing pre-metastatic niche formation [87]. Furthermore, an inhibitor of the SDF-1 receptor CXCR4 and anti-CXCR4 antibody treatment was proven to reduce SDF-1-mediated neutrophil recruitment to the liver, and its interaction with TIMP1 could contribute to the formation of the pre-metastatic niche [88]. However, the explicit mechanisms of pre-metastatic niche formation in PDAC have yet to be determined.

**Figure 2:**
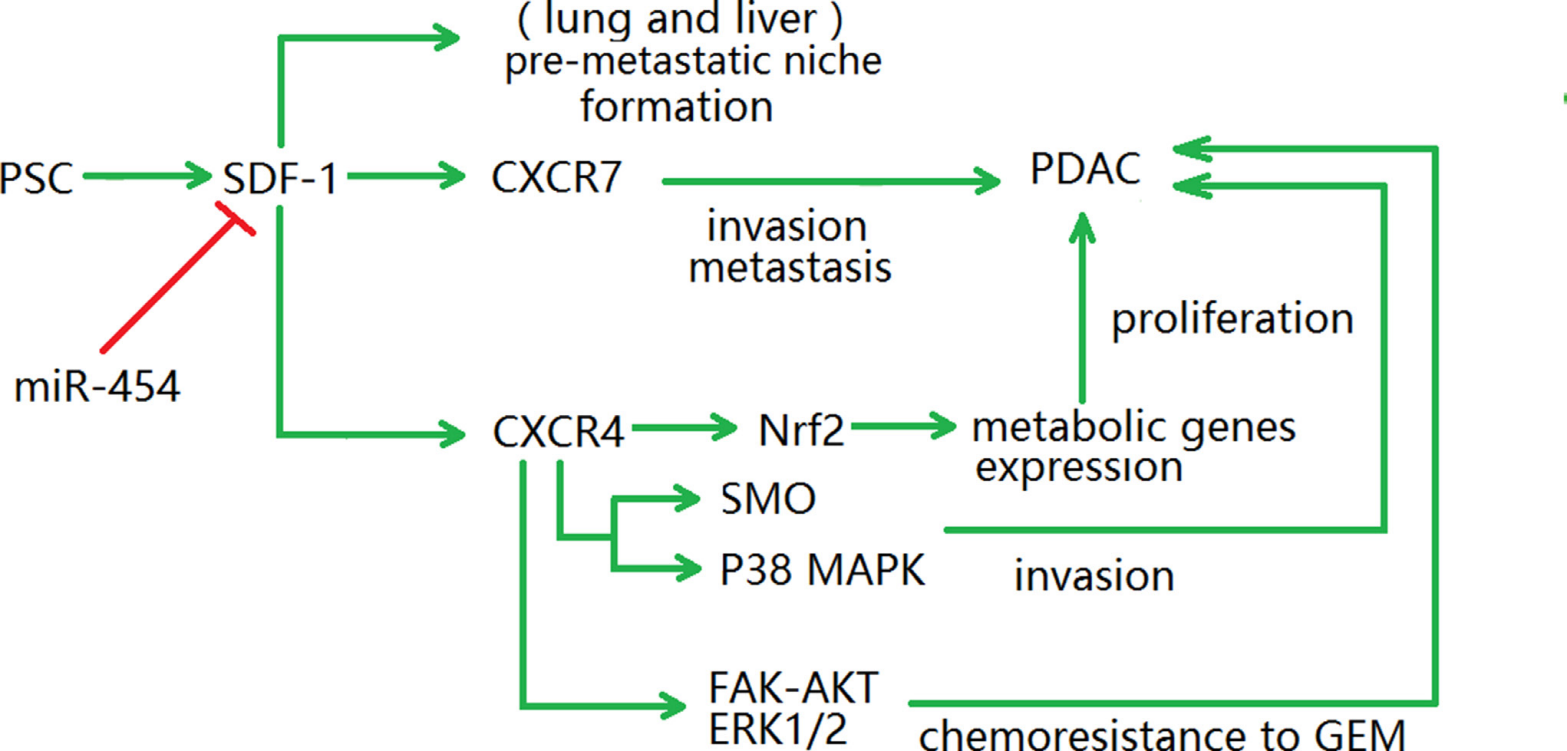
Diagram illustrating the SDF-1 signaling pathways There are several SDF-1 signaling pathways involving with the malignant development of PDAC, which play the role in invasion, metastasis, metabolic genes expression and chemoresistance to GEM. In addition, SDF-1 participates in the formation of pre-metastatic niche in lung and liver cancers, but this process is not yet clear in PDAC. NRF2, nuclear factor erythroid 2; SMO, Smoothened; FAK, focal adhesion kinase; ERK, extracellular signal-regulated protein kinases; GEM, gemcitabine.

There are several signaling pathways of SDF-1-induced invasion and metastasis in PDAC, and their mechanisms are diverse [89]. A recent study found that a SDF-1-targeting miRNA, miR-454, plays a role in TAM recruitment, which has value for understanding the mechanisms underlying tumor growth [90]. Some studies have shown that high SDF-1 expression levels are related to poor outcome of PDAC [91]. Interestingly, for many years, it was assumed that CXCR4 was the only SDF-1 receptor; however, recent studies have demonstrated that CXCR7 is a novel receptor of SDF-1 involved in several aspects of tumor invasion and metastasis in PDAC [81, 91, 92]; Liu et al. reported that SDF-1 and CXCR7's expression in the ductal cells were related with poor prognosis, as the median survival time of SDF-1^+^CXCR7^+^ patients was 6 months while that of SDF-1^−^CXCR7^−^ patients was 10 months [81]. In addition, there is cross-talk between the SDF-1 subtype SDF-1α and IL-6 signaling, which promoted tumor cell proliferation and chemoresistance in PDAC [84]. Moreover, a recent study asserted that PSC-secreted IL-6 and SDF-1α were responsible for nuclear factor erythroid 2 (NRF2) activation in PDAC cells. The expression of metabolic genes was upregulated by NRF2, which then promoted the detoxification of ROS and the synthesis of purine nucleotides, leading to PDAC cell proliferation [84, 93]. Another meaningful cross-talk is between the SDF-1/CXCR4 axis and the non-canonical Hh pathway [94]; the latter was usually considered to function in developing embryos [95]. It has been illustrated that the SDF-1/CXCR4 axis could promote the expression of smoothened, a vital protein in the Hh pathway whose overexpression can induce tumor cell EMT and invasion. To conclude, the cross-talk between SDF-1 and other signaling pathways provides a novel platform for deeper understanding of the mechanisms involved in SDF-1-induced invasion and metastasis in PDAC (Figure 2).

### PSC-secreted IL-6 and potential targeted therapies

In PDAC microenvironment, IL-6 is one of the most abundant proinflammatory cytokines secreted by PSCs and tumor cells. In addition to the inflammatory response, IL-6 is associated with numerous tumor cell biological behaviors, including growth, survival, metastasis, angiogenesis, EMT, and chemoresistance [84, 97], which plays a role in the promotion of pancreatic cancer development. Recent research has shown that several IL-6 pathways regulated the relationship between PSCs and PDAC cells and specific inhibitors or antagonists could block the pathways for targeted therapy. Previous studies have stated that elevated MDSC levels were associated with reduced overall survival in patients with pancreatic cancer [97], and IL-6 produced by PSCs in TME could activate MDSC via JAK/STAT3 (Janus kinase/signal transducer and activator of transcription 3) signaling [98]. The activated MDSCs play a role in immunosuppression in the TME, which can protect PDAC cells from the immune cells’ attack and this could explain why high MDSC levels are usually related to poor prognosis. Recently, Hamada et al. found that the STAT3 pathway also regulated PSC-induced EMT in pancreatic cancer cells, [96, 99]. Furthermore, the NRF2 pathway was demonstrated to be induced by STAT3 to promote EMT in PDAC [96]. In short, IL-6/JAK/Stat3/NRF2 signaling pathway is a valuable target to seek suitable therapy options for PDAC patients. In addition, it has been proven that IL-6 is involved in the activation of macrophage phenotype switch, which takes place in the TME, followed by stimulated expression of EMT markers such as N-cadherin and vimentin in pancreatic cancer cells [100]. Finally, recent study argued that PSC-derived IL-6 was regulated by autophagy, and persistent PSC activation might be caused by an autocrine IL-6 loop [18]. In summary, further researches involving IL-6 in PDAC should focus on: 1). IL-6-related inflammatory response, 2). IL-6/JAK/Stat3/NRF2 signaling pathway, 3). IL-6-induced EMT process and the activation of macrophage phenotype switch, 4). IL-6-related autophagy.

All of the above forms a solid foundation for treating PDAC by targeting IL-6. Currently, the value of clinically available small molecules such as trametinib, regorafenib, sorafenib, and sunitinib as antineoplastic drugs is reflected in the suppression of the expression of IL-6 or IL-6 mRNA [101, 102]. However, these drug experiments were based on mouse models and require further investigation in the future. Table 3 lists some novel potential therapies targeting IL-6 pathways that are promising for treating PDAC.

**Table 3:** The potential therapies targeting IL-6 pathways for treatment of PDAC

Agent or candidate target	Descriptions	Impacts	mechanism
Saha [100]	Histone Deacetylase HDAC I/II inhibitor	prevented neoplastic lesion formation, fbrosis, and M2 macrophage in the KC mice exposed to cigarette smoke.	inhibited HDAC3 to decrease the level of IL-6 produced by the cancer cells.
JQ1 and I-BET 762 [148]	Bromodomain inhibitors, small molecule inhibitors that target BET (bromodomain and extra terminal) proteins	decreased c-Myc and p-Erk 1/2 protein levels and inhibited proliferation in pancreatic cancer cells.	suppressed the production of nitric oxide and inflammatory cytokines including IL-6.
GV1001 [149]	a telomerase-based cancer vaccine	the combination treatment of GV1001 and gemcitabine could signifcantly reduce the fbrosis in tumor tissue and induce apoptosis.	the combination can suppress the IL-6, however, the actual mechanism remains to be investigated.
HIC1 [150]	Hypermethylated in cancer 1 ,a tumour suppressor gene	negative HIC1 expression predicted poor dignosis; inhibited the invasion and metastasis of pancreatic cancer cells both *in vitro* and *in vivo*; repressed the expression of STAT3 target genes, including c-Myc, VEGF, CyclinD1, MMP2 and MMP9.	inhibited STAT3 activity and it was likely to function via inhibition of IL-6/JAK/STAT3 signaling pathway.
ANXA2 [151]	Annexin A2,a negatively charged phospholipidbinding protein	mediated resistance to gemcitabine.	increased the activity of NF-κB, whose downstream target genes including that encoding IL-6.
RA [152]	Retinoic acid, a small molecular derivative of vitamin A	inhibited pancreatic cancer cell migration and EMT.	reduced IL-6 secreted by CAFs(cancer associated fibroblasts).

### Galectin-1 functions as a tumor promoter in PDAC

A 29-kDa β-galactoside-binding protein, galectin-1 is a member of the galectin lectin family. It functions both inside and outside the cell; however, its carbohydrate-binding role is extracellular [103, 104]. And the extracellular effects of galectin-1 in PDAC progression will be discussed in this section. The previous literature has shown that galectin-1 was secreted by activated PSCs in PDAC stroma [105]. In the TME, persistent PSC activation promotes tumor cell malignant behavior, and galectin-1 plays a crucial role in PSC activation [27] [106].

Recent studies have demonstrated that chemokine production, PSC proliferation, collagen and fibronectin synthesis could be induced by galectin-1 [107, 108], and they were responsible for the tumor desmoplastic reaction around cancer cells [109]. Moreover, these effects could lead to immunosuppression in PDAC [106]. In a healthy human body, the immune system maintains a balance between tumor immunosuppression and anti-tumor activity [110]. However, in PDAC, galectin-1 could help tumor cells to escape from immune surveillance [106]. The actual function profile of the immunosuppressive is that galectin-1 can induce effector T cell apoptosis and anergy via the “caspase first” or “mitochondria first” pathways and alter Th1/Th2 balance by stimulating Th2 cytokine (IL-6 and IL-10) secretion but decreasing Th1 cytokine (tumor necrosis factor-β and interferon-γ) secretion [106]. Moreover, tumor-related process such as invasion, angiogenesis, proliferation, MMP2, MMP9 expression, and EMT process could be induced by galectin-1 in PDAC [51, 104, 111, 112]. Acinar–ductal metaplasia (ADM) is a significant process in PDAC development, which is triggered by PSC-secreted galectin-1 via the epidermal growth factor receptor and pancreatic and duodenal homeobox 1 pathways [113]. In addition, the Hh/Gli axis could be another pathway involved in ADM, but its mechanism has not been elucidated. As described hereinbefore, SDF-1 is a critical tumor–stromal interaction mediator and could promote PDAC progression. It was proven to be upregulated by endogenous galectin-1, promoting tumor metastasis [82]. Furthermore, PSC-secreted galectin-1 is upregulated by TGF-β1, which is produced by PDAC cell, and TGF-β1 can stimulate PSC activation simultaneously, inducing more galectin-1 secretion. The malignant behavior of PDAC is related to this vicious cycle of mutually reinforcing mechanism [112].

**Figure 3:**
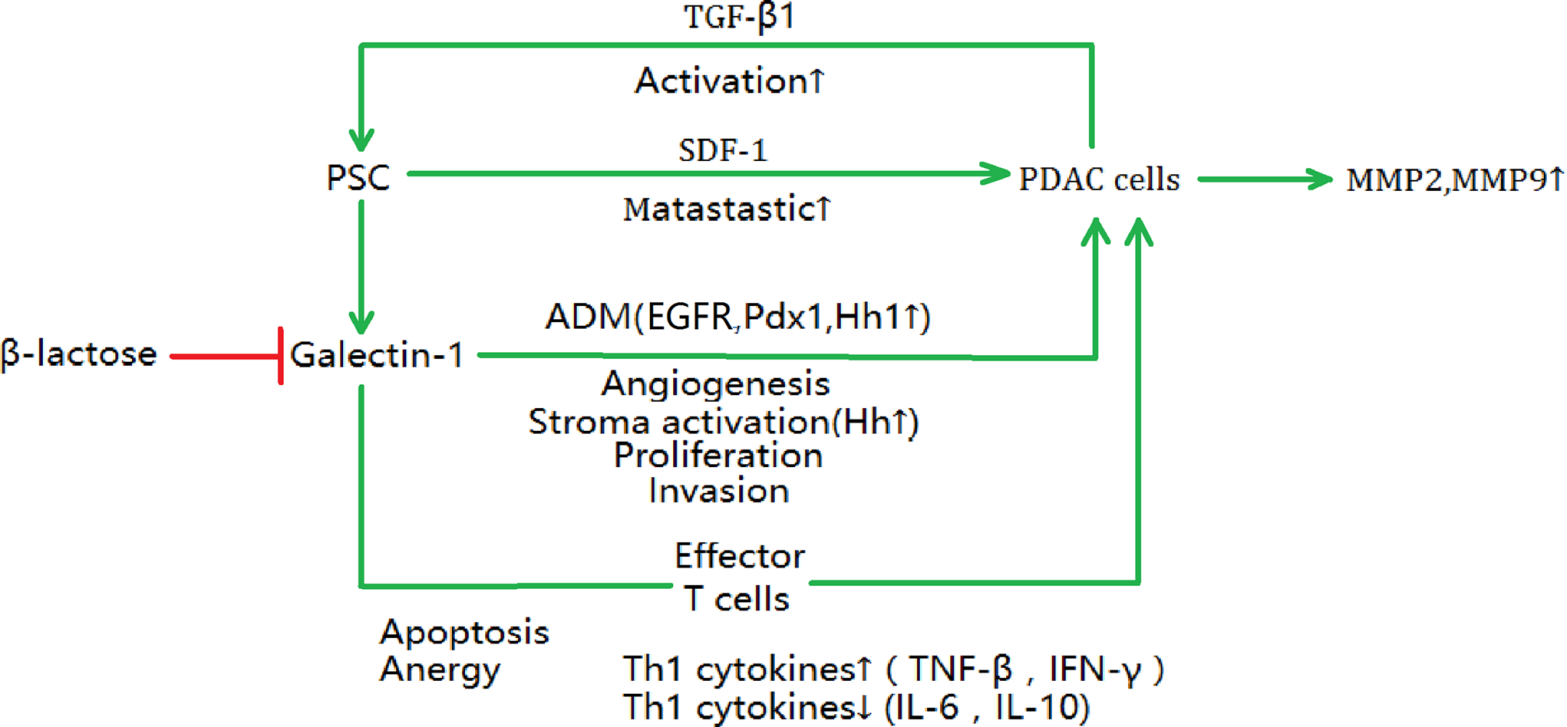
The effects of PSC-induced galectin-1 on PDAC cell progression There are various extracellular effects of galectin-1 in PDAC progression, such as metastasis, ADM (acinar–ductal metaplasia), angiogenesis, stroma activation, proliferation, invasion of PDAC cells. In addition, the galectin-1 contributes to the apoptosis and anergy of effector T cell in immune system and alters Th1/Th2 balance. SDF-1, stromal cell-derived factor-1; TGF-β, transforming growth factor β; EGFR, epidermal growth factor receptor; Pdx1, pancreatic and duodenal homeobox 1; Hh1, hedgehog1; IFN-γ, interferon-γ; IL-6, interleukin 6; IL-10, interleukin 10.

There is a positive association between galectin-1 expression and PDAC tumor size, lymph node metastasis, perineural invasion, differentiation, Union for International Cancer Control stage, and survival [106, 112, 114]. As various promoters influence galectin-1 expression during tumor formation, galectin-1 might be a promising drug target and biomarker for PDAC [115, 116]. These valuable preclinical evidence showed that inhibiting galectin-1 could be efficient for treating PDAC. For example, β-lactose, a competitive inhibitor of galectin-1, could inhibit the immunosuppressive effect induced by galectin-1 *in vivo* [106]. In addition, monoclonal antibodies are a promising therapeutic approach, e.g., 0118 (PTX008, OTX008), as they have high specificity for galectin-1 and are small molecules [116]. However, most of the galectin-1-targeting therapies are in preclinical or early clinical development and their prolonged clinical application remain far on the horizon (Figure 3).

### HGF: a novel target for PDAC treatment

HGF is a 90-kDa glycoprotein, mainly originating from stromal cells such as blood endothelial cells, macrophages, fat-storing cells, neutrophils, and fibroblasts [117]. In PDAC, HGF is mainly produced by PSCs and plays a critical role in the cross-talk between the PDAC cells and PSCs [118–120]. The various functions of HGF are realized mainly via binding to its specific tyrosine-kinase receptor c-MET [121, 122], and the HGF-c-MET pathway recently has become a research hotspot involving PDAC progression. Upon binding to HGF, c-MET could activate its multiple downstream pathways, such as PI3K/Akt, MAPK, and STAT3, promoting invasion, proliferation, migration, mitogenesis, DNA-synthesis and chemotherapy resistance of PDAC cells [123–125].

In PDAC, the angiogenesis is a crucial process involving tumor growth, progression, and metastasis, which could be evaluated by the proliferation of vascular endothelial cell and the formation of tube [125, 126]. Vascular endothelial growth factor (VEGF) is a well-known pro-angiogenic growth factor, and the inhibitor of VEGF and its receptor have been approved by the FDA (Food and Drug Administration, USA) in PDAC treatment [127]. Nevertheless, recent researches showed VEGF inhibitors could promote the metastasis and invasion of tumor cell, followed by decreased survival [128–130]. Interestingly, the anti-HGF therapy showed its edge when conflicting with PDAC. Patel MB et al. demonstrated that targeting HGF/c-MET and urokinase-type plasminogen activator (uPA) pathways could be beneficial for inhibiting endothelial cell proliferation and closed tube formation [125]. AMG102 and amiloride, the specific inhibitors of HGF and uPA, were proven to have potent antiangiogenic influences when combined, and they were independent of patient heterogeneity, which has more practical and clinical value. However, not all the combination therapy could achieve expected outcome, and we should figure out the exact mechanism underlying [131–133]. As illustrated before, gemcitabine is a standard chemotherapeutic agent for PDAC. When combined with HGF inhibition, the antimetastatic, antiangiogenic and antiproliferative effects were reduced and there have even been cases of tumor progression [119]. This could be explained by the theory that gemcitabine can select out treatment-resistant and aggressive cancer cells, and could promote EMT process and metastasis of cancer cells [119, 134].

When using HGF inhibition therapy, we should take functional heterogeneity into consideration as well. The p53 gene is an anti-oncogene whose mutation is related to more than half of human cancer [135]. Yang et al. found that P53 deficiency could increase the invasion and migration of Panc-1 cells, which could upregulate the expression of c-MET [120]. Furthermore, a member of the inhibitor of apoptosis protein, Survivin, was proven to be upregulated by HGF-c-MET pathway, which could promote the tumor progression. However, in the same research, another cell line SW1990 showed low sensitivity to HGF, and that could be explained by the difference of the expression level of c-MET in two cell lines. This finding suggested that c-MET could be a novel biomarker for choosing suitable patients who can benefit the most from the treatment targeting HGF. Similarly, the various PSC populations were proven to be heterogeneous, and their capacity to stimulate the migration and DNA-synthesis of PDAC cells and expression level of HGF were different according to the research based on eight different PDAC patients [124], which was in agreement with the previous study about heterogeneity of PSCs [136]. Furthermore, IL-1α and TGF-β were demonstrated to play a role in regulating the expression of HGF secreted by PSC and the multifunction induced by HGF. Recent studies demonstrated that approximately 50% TGF-β signaling pathway exists mutations [137, 138], and the heterogeneity of TGF-β could contribute to functional heterogeneity of PSCs. As the complexity of various signaling pathways between PSCs and PDAC cell is further complicated by the diverse expression level among different patients, inhibiting HGF could have different outcomes in different subsets of patients.

### Outlook: The inhibition of PSC-derived soluble factors is promising for PDAC treatment

The interaction between PSCs and PDAC cells play an important role in the promotion of tumor development. Nowadays, there are increasing attentions on researches of the cytokines or proteins in PDAC microenvironment, i.e., TGF-β, IL-6, SDF-1, HGF and galectin-1, which have their own regulatory downstream signaling pathways of PSCs and PDAC cells. Furthermore, there are usually cross-talks between these cytokines or proteins, such as between IL-6 and SDF-1α, which has a synergistic effect during PDAC formation. Intervening in these pathways and blocking their critical processes could be valuable for treating PDAC. However, it needs more work to clarify the definite mechanisms of these pathways, and to discover more effective targeting agents.

## Abbreviations

α-SMA: α-smooth muscle actin
ADM: acinar–ductal metaplasia
BMP7: bone morphogenetic protein 7
CAF: carcinoma-associated fibroblasts
COX-2/EP3: cyclooxygenase 2/prostaglandin E receptor 3
CSC: cancer stem cell
ECM: extracellular matrix
EGFR: epidermal growth factor receptor
EMT: epithelial–mesenchymal transition
FGF2: fibroblast growth factor 2
FOXP3: forkhead box 3
GFAP: glial fibrillary acidic protein
GM-CSF: granulocyte/macrophage colony-stimulating factor
Hh: Hedgehog
IFN-γ: interferon-γ
IL-1: interleukin 1
IL-6: interleukin 6
IL-8: interleukin 8
JAK/STAT3: Janus kinase/signal transducer and activator of transcription 3
KLF5: Krüppel-like factor 5
MAPK: mitogen-activated protein kinase
MCP-1: monocyte chemoattractant protein-1
MDSC: myeloid-derived suppressive cell
MET: mesenchymal–epithelial transition
miRNA: microRNA
MMP: matrix metalloproteinase
NF-κB: nuclear factor-κB
NOX4: NADPH oxidase 4
NRF2: nuclear factor erythroid 2
OV: oncolytic virus
PDAC: pancreatic ductal adenocarcinoma
PDX1: pancreatic and duodenal homeobox 1
PSC: pancreatic stellate cell
PDGF: platelet-derived growth factor
ROS: reactive oxygen species
SDF-1: stromal cell-derived factor-1
SHH: Sonic Hh
SMO: Smoothened
TAM: tumor-associated macrophage
TAp73: transcriptionally active p73
TGF-β: transforming growth factor β
TIMP: tissue inhibitors of MMP
TME: tumor microenvironment
TNF-β: tumor necrosis factor-β
Treg: regulatory T cells
UICC: Union for International Cancer Control
EFGR: Epidermal growth factor receptor
uPA: urokinase-type plasminogen activator
